# Galanin Protects from Caspase-8/12-initiated Neuronal Apoptosis in the Ischemic Mouse Brain via GalR1

**DOI:** 10.14336/AD.2016.0806

**Published:** 2017-02-01

**Authors:** Yun Li, Zhu Mei, Shuiqiao Liu, Tong Wang, Hui Li, Xiao-Xiao Li, Song Han, Yutao Yang, Junfa Li, Zhi-Qing David Xu

**Affiliations:** Department of Neurobiology and Beijing Institute for Brain Disorders, Capital Medical University, Beijing 100069, China; Department of Neurobiology and Beijing Institute for Brain Disorders, Capital Medical University, Beijing 100069, China; Department of Neurobiology and Beijing Institute for Brain Disorders, Capital Medical University, Beijing 100069, China; Department of Neurobiology and Beijing Institute for Brain Disorders, Capital Medical University, Beijing 100069, China; Department of Neurobiology and Beijing Institute for Brain Disorders, Capital Medical University, Beijing 100069, China; Department of Neurobiology and Beijing Institute for Brain Disorders, Capital Medical University, Beijing 100069, China; Department of Neurobiology and Beijing Institute for Brain Disorders, Capital Medical University, Beijing 100069, China; Department of Neurobiology and Beijing Institute for Brain Disorders, Capital Medical University, Beijing 100069, China; Department of Neurobiology and Beijing Institute for Brain Disorders, Capital Medical University, Beijing 100069, China; Department of Neurobiology and Beijing Institute for Brain Disorders, Capital Medical University, Beijing 100069, China

**Keywords:** Apoptosis, Receptors, Stroke, Neuroprotection, Neurotransmitters

## Abstract

Galanin (GAL) plays key role in many pathophysiological processes, but its role in ischemic stroke remains unclear. Here, the models of 1 h middle cerebral artery occlusion (MCAO)/1-7 d reperfusion (R)-induced ischemic stroke and *in vitro* cell ischemia of 1 h oxygen-glucose deprivation (OGD)/24 h reoxygenation in primary cultured cortical neurons were used to explore GAL’s effects and its underlying mechanisms. The results showed significant increases of GAL protein levels in the peri-infarct region (P) and infarct core (I) within 48 h R of MCAO mice (*p*<0.001). The RT-qPCR results also demonstrated significant increases of *GAL* mRNA during 24-48 h R (*p*<0.001), and GAL receptors *GalR1-2* (but not 3) mRNA levels in the P region at 24 h R of MCAO mice (*p*<0.001). Furthermore, the significant decrease of infarct volume (*p*<0.05) and improved neurological outcome (*p*<0.001-0.05) were observed in MCAO mice following 1 h pre- or 6 h post-treatment of GAL during 1-7 d reperfusion. GalR1 was confirmed as the receptor responsible for GAL-induced neuroprotection by using GalR2/3 agonist AR-M1896 and Lentivirus-based RNAi knockdown of GalR1. GAL treatment inhibited Caspase-3 activation through the upstream initiators Capsases-8/-12 (not Caspase-9) in both P region and OGD-treated cortical neurons. Meanwhile, GAL’s neuroprotective effect was not observed in cortical neurons from conventional protein kinase C (cPKC) γ knockout mice. These results suggested that exogenous GAL protects the brain from ischemic injury by inhibiting Capsase-8/12-initiated apoptosis, possibly mediated by GalR1 via the cPKCγ signaling pathway.

Stroke is the leading cause of morbidity and mortality. According to statistics gathered by the American Heart Association, about 795,000 people experience a new or recurrent stroke every year and a person dies from a stroke every 4 minutes on average [1]. So far, treatments are almost entirely dependent on timely reperfusion and using rehabilitation therapies to control the post-stroke neurological deficits, such as motor and sensory motor deficits. Although the thrombosis time window has been prolonged to 4.5 h, much work still remains to be done in attempting to improve post-stroke outcome [2]. Recent studies made on the perceived quality of life (QoL) and psychological well-being of stroke survivors show an impaired QoL and various comorbidities, such as anxiety, depression, and raised brain natriuretic peptide levels [3]. Thus, even if a person survives the initial onset of ischemic stroke, he/she is still unlikely to attain a full recovery.

Numerous studies have unraveled molecules and pathways that mediate the process of cell death following ischemia. Among these are studies that focus on neuropeptides [4-7]. Galanin (GAL), a peptide made up of 29 amino acids (30 in humans), was first discovered in the porcine intestine and has a wide distribution in the central and peripheral nervous system [8-10]. It has also been reported that GAL regulates numerous physiological actions, such as neuroendocrine regulation, mood, cognition, energy and osmotic homeostasis [10]. Thus, galaninergic signaling is involved in a large number of disease states, including chronic pain, epilepsy, mood disorders, Alzheimer’s disease and addiction. It has been shown that GAL regulates its physiological and pathophysiological processes by interacting with its three G protein-coupled receptors, GalR1, GalR2, and GalR3 [10]. There is also evidence suggesting a role in neuroprotection [8-10]. For example, GAL has been observed to have neuroprotective roles in many cases, such as beta-amyloid poisoning [11-13], glutamate induced excitotoxicity [14], high glucose induced apoptosis [15], and shear stress induced injury [16].

GAL’s ability to protect the neural system during injuries suggests that it has the potential to be used in stroke treatment. Earlier studies have shown up-regulation of GalR1 mRNA in the rat cerebral cortex at 24 h following transient focal cerebral ischemia [17] and in the locus coeruleus 7 d after 60-min transient MCAO [18]. However, the role of GAL in ischemic stroke is still unclear. In this study, the intraluminal MCAO mouse model was performed to determine the effects that GAL would have on the outcome of stroke. At the same time, the cell model of ischemic injury *in vitro* by way of subjecting primary cultured cortical neurons to oxygen-glucose deprivation (OGD) was used to explore the underlying workings of GAL and its receptors during ischemic injury.

## MATERIALS AND METHODS

All chemicals used for sodium dodecyl sulfate (SDS) polyacrylamide gel electrophoresis (PAGE) and 2,3,5-triphenyltetrazolium chloride (TTC) staining were purchased from Sigma-Aldrich (St. Louise, MO 63103, USA). Adult male C57BL/6J wild type (WT) and conventional protein kinase C (cPKC)γ knockout mice, at 8-10 weeks of age (weighing 20-25 g), were purchased from The Jackson Laboratory (Bar Harbor, ME 04609, USA), and maintained and genotyped in the Experimental Animal Center of Capital Medical University, PR China. They were housed in groups of five under constant temperature (23±2°C) and maintained on a 12-h light/dark cycle with food and water available *ad libitum*. All procedures were performed in accordance with guidelines set by the Animal Care and Use Committee of Capital Medical University and ARRIVE (Animal Research: Reporting *In Vivo* Experiments), and the procedures were also consistent with the NIH Guide for the Care and Use of Laboratory Animals (NIH Publications No. 80-23).

### Transient MCAO-induced Ischemic Stroke Mouse Model

Surgery was conducted at room temperature (18-22°C) on C57BL/6J mice and the ischemic stroke mouse model was performed as described previously [19-21] with a slight difference in the model used here being a transient occlusion followed by reperfusion instead of the permanent MCAO mentioned in the cited publications from our laboratory. In brief, the mice were laid out on a surgery platform and a ventral midline incision was made to expose the common carotid artery (CCA) after anesthetizing with sodium pentobarbital (60 mg/kg, i.p.). Upon full exposure of the CCA, it was temporarily ligated to prevent hemorrhage. Thereafter, the external carotid artery (ECA) was exposed and a permanent ligation was made as distally as possible, a loop was also made closer to the bifurcation. Blood flow to the internal carotid artery (ICA) was then restricted using a vascular clip. With a pair of microscopic scissors, a small incision was made on the ECA, and a 4-0 surgical nylon monofilament with its tip (0.23 mm in diameter) rounded by heat was inserted to the ICA. While moving the monofilament forward to a point of approximately 12 mm, mild resistance was felt, suggesting that it has reached the origin of the middle cerebral artery (MCA). After the MCA was occluded for 1 h, the monofilament was removed and the ECA permanently ligated to prevent bleeding from the incision. Finally, reperfusion was achieved by loosening the temporary ligation on the CCA. Post-operative mice were placed in a heated cage for a minimum of 2 h. For monitoring cerebral blood flow (CBF) during MCAO surgery and GAL injection, Laser Doppler Flowmetry was conducted with the PeriFlux 5000 system (Perimed, Jarfalla Stockholm, Sweden). Regional CBF decreased by 80% in mice after MCAO, and recovered completely after the monofilament was removed 1 h later.

### Intracerebroventricular Injections of Galanin

Mice were randomly divided into three groups: Sham, MCAO and GAL pre-treatment + MCAO groups. The neuropeptide GAL was diluted with saline to obtain concentrations of 1 μmol/L and 1 nmol/L, of which 5 μl of either concentration was injected 1 h before MCAO surgery. The intracerebroventricular (i.c.v) injection procedure followed that of a previous report [21]. Mice were anesthetized with sodium pentobarbital (0.06 g/kg) and placed in a stereotaxic frame. The cannula (28 gauge, inner diameter 0.18 mm, outer diameter 0.36 mm) was lowered into the right cerebral ventricle using the following coordinates: 0.5 mm posterior to bregma, 1.0 mm lateral to bregma, and 3.5 mm below the skull surface.

For post-treatment with GAL after MCAO surgery, a cannula (28 gauge, inner diameter 0.18 mm, outer diameter 0.36 mm) was inserted into the brain at the coordinates described above and fixed at that position with medical glue. After the procedure, mice were returned to their cages and allowed to recover for 7 d, by which time the MCAO surgery was conducted and a single dose of GAL at the concentration of 1 μmol/L was given via the cannula 6 h after reperfusion.

### Evaluation of Neurological Deficits

Mice were tested at 24 h, 72 h and 7 d after reperfusion. Neurological deficits were measured using the simplified procedure created by Rodriguez and colleagues [22], with a maximum score of 10 wherein the mouse shows the most deficits and a minimum score of 0 where there are no neurological deficits present. The detailed criteria were listed as follows: 0, no neurological dysfunction; 2, slight dysfunction in mobility and presence of passivity; 4, moderate neurological dysfunction; 6, more handicapped animals with more marked hypomobility, circling, tremor, jerks and/or convulsions, forelimb flexion and moderate motor incoordination; 8, respiratory distress and total incapacity to move/coordinate; and 10 refers to death due to 1 h MCAO/1-7d reperfusion. If the level of deficits didn’t meet the precise criteria, the nearest appropriate number 1, 3, 5, 7 or 9 were recorded.

### Evaluation of Muscle Strength and Limb Coordination

The 1 h MCAO mice were tested at 24 h, 72 h and 7 d after reperfusion during which the mouse was placed upside down on a steel grid suspended 35 mm above ground with soft bedding underneath to break its fall. The time until the mouse completely releases its grasp and falls down is recorded. A score of 0 was given to mice that fell immediately. 600 s was the time out period. Each mouse was tested twice on every testing day and the average time was calculated.

To test limb coordination, we used an adapted version of the cylinder test that was first described by Li et al [23]. Briefly, mice were placed inside a clear cylinder about 9-cm in diameter and 15-cm in height. A mirror was placed behind the cylinder to enable the observer to record forelimb movements when the mouse was turned away from the observer. After the mouse was put into the cylinder, forelimb contact against the wall after rearing and during lateral exploration was recorded by following the criteria described by Li et al [23]. The final score was recorded as (non-impaired forelimb movement - impaired forelimb movement)/(non-impaired forelimb movement + impaired forelimb movement + both movements).

### Measurements of Infarct Volume and Edema

For TTC staining, the 1 h MCAO mice were sacrificed at 24 h reperfusion. The brain was isolated and cut into 1.5 mm thick coronal sections (five sections from one brain). Brain sections were incubated for 20 min in a solution of 0.5% TTC (Sigma-Aldrich, St. Louise, MO 63103, USA) in 10 mM phosphate buffered saline at 37 °C. The images of stained slices were scanned into a computer. As reported by Wexler et al [24], the infarct volume was determined using Image Pro Plus 6.0 (Media Cybernetics, Rockville, MD 20852, USA). Edema ratio (E) was calculated using the equation: E = (ΣVR-ΣVL)/(ΣVL + ΣVR)×100%; ΣVR and ΣVL are the volume of right (ischemic) and left (non-ischemic) hemisphere, respectively. Background was calculated using the following equation: B = ΣVS/ΣVT×100%, where ΣVS is the volume of the unstained white matter in the sham group and ΣVT is the total brain volume. Considering the effect of edema and background, infarct volume was determined indirectly and represented as a percentage of the entire brain using the equation: I = [ΣVI×(1-E)/ΣVT×(1-B)]×100%, where ΣVI is the volume of tissue that is not stained with TTC in MCAO mice.

### Western Blot Analysis

Mouse brains were removed at 24, 48 and 72 h reperfusion after 1 h MCAO and placed into ice-cold artificial cerebral spinal fluid (ACSF, in mM: NaCl 125.0, KCl 2.5, CaCl_2_ 2.0, NaHCO_3_ 26.0, NaH_2_PO_4_ 1.25, MgCl_2_ 1.0, glucose 5.0, pH 7.4). According to previous reports [25, 26], cerebral cortex from areas of the ischemic core (I), peri-infarct region (P) and contralateral non-ischemic region (C) were collected. In brief, the area at 2 mm from the anterior tip of the frontal lobe were cut, and the ipsilateral hemisphere was sectioned to four 2 mm thick slices, after which the tissue supplied by the anterior cerebral artery was removed with a longitudinal cut approximately 1 mm from the midline. The I and P regions were then separated by a transverse diagonal cut at approximately a 45° angle. The corresponding regions from the non-ischemic hemisphere were collected as contralateral control. The samples were frozen in liquid nitrogen, and kept at -80 °C for analysis at a later time.

As reported previously [27], the tissue was thawed and homogenized with Buffer C, which is Buffer A [50mM Tris-Cl, pH 7.5, containing 2 mM EDTA, 1 mM EGTA, 100 mM iodoacetamide (SH-group blocker), 5 μg/mL each of leupeptin, aprotinin, pepstatin A and chymostatin, 50 mM potassium fluoride, 50 nM okadaic acid, 5 mM sodium pyrophosphate] containing 2% SDS. Protein concentration was determined via BCA kit (Pierce Company, Rockford, IL 61101, USA), using albumin dissolved in Buffer C as standards.

A total of 50 μg from each sample was loaded onto 12% gels. The gels were electrophoresed then transferred onto polyvinylidenedifluoride (PVDF) membranes (Millipore, Billerica, MA 01821, USA) at 4°C. After rinsing with TTBS (20 mM Tris-Cl, pH 7.5, 0.15 M NaCl and 0.05% Tween-20), the transferred PVDF membranes were blocked with 10% non-fat milk for 1 h. The primary rabbit antibody against GAL (Millipore, Billerica, MA 01821, USA) was incubated overnight (4 °C) at a dilution of 1:2000. For analysis of Caspases and LC3 hydrolysis, the primary antibodies used at a dilution rate of 1:1000 were as follows: monoclonal antibodies against Caspase-3 (#9665), Caspase-8 (#8592), Caspase-9 (#9508) and Caspase-12 (#2202) were purchased from Cell Signaling Technology (CST, Danver, MA 01923, USA). As an internal reference, the membrane was also incubated for 3 h at RT with the primary mouse monoclonal antibody against β-actin (Proteintech, Chicago, IL 60612, USA). Horseradish peroxidase-conjugated goat anti-rabbit or anti-mouse IgG (The Jackson Laboratories, Bar Harbor, Maine 04609, USA) were used as secondary antibodies for a hour incubation at 1:5000 dilutions. The enhanced chemiluminescence kit (GE Healthcare, Buckinghamshire, UK) was used to detect the signals.

### RT-qPCR Analysis

The extraction process of cerebral tissue samples was similar to that described in the previous section. After retrieving the frozen tissue from -80 °C, the samples were placed in QIAzol Lysis Reagent (QIAGEN Cooperation, 22525 Hamburg, Germany) and homogenized. RNA was extracted using the RNeasy Lipid Tissue Mini Kit (QIAGEN Cooperation) and reverse transcribed into cDNA using Transcriptor First Strand cDNA Synthesis Kit (0489703001, Roche Life Sciences, Indianapolis, IN 50414, USA). The Power SYBR Green PCR Master Mix (Life Technologies, Carlsbad, CA 92008, USA) was used to perform RT-PCR with the iQ5 optical system software (Bio-Rad Laboratories, Hercules, CA 94547, USA) according to the manufacturer’s protocol. Specific primers used are shown as follows: GAL (Forward: 5′-CACTCTGGGACTTGGGATG-3′; Reverse: 5′-CAGGC AAGAGGGAGTTACAA-3′); GalR1 (Forward: 5′-CTTACTGCTCATCTGCTTTTGC-3′; Reverse: 5′-GTAT TTGGCATATCCTGGCTG-3′); GalR2 (Forward: 5′-CCTCATCTTCCTCACTATGCAC-3′; Reverse: 5′-CAT CGGGCTCATCTGGG-3′); GalR3 (Forward: 5′-AGGATGAAGCAAAGGTCGG-3′; Reverse: 5′-CAAC AGGAAGATGAGGGCAA-3′); and Ywahz (Forward: 5′-GAAGACGGAAGGTGCTGAG-3′; Reverse: 5′-GACT TTGCTTTCTGGTTGCG-3′). Relative gene expression was normalized using the housekeeping gene Mus musculus tyrosine 3-monooxygenase/tryptophan 5-monooxygenase activation protein zeta polypeptide (*ywahz*) [28]. Experiments were repeated in triplicates and the results were calculated by the 2^-ΔΔCt^ method.

### GAL Receptor Knockdown via Lentiviral Vector

The GalR1 and GalR2 knockdown Lentivirus was purchased from Genechem (Shanghai, PR China) and administered in both *in vivo* and *in vitro* tests to ascertain the role of GalR1 as the main mediator of GAL’s protective effects. The sequence of the anti-GalR1 siRNAi 5′-GGTTCTCAATCATCTGCAT-3′, and for negative control, the lentivirus vector containing the scrambled sequence of 5′-GATCCTCTTATG GCACTTA-3′ was used. Sequence of the anti-GalR2 is 5′-AGGTGACACGGATGATCAT-3′, with a scrambled sequence of 5′-GGCAAAGGCTACGACATTGTA-3′ used as control.

For *in vivo* studies, the lentivirus was injected 5 d before i.c.v injection of GAL and MCAO surgery using the same protocols mentioned by Wang et al [29]. Briefly, the mice were anesthetized with sodium pentobarbital (0.06g/kg) and put on a stereotaxic instrument, 1 μl of Lentivirus suspension liquid containing 1 × 10^8^ TU/ml was injected into each point (point 1: 0.3 mm anterior to bregma, 3.0 mm lateral to bregma and 2.0 mm below the skull surface; point 2: 0.8 mm posterior to bregma, 3.0 mm lateral to bregma and 2.0 mm below the skull surface; point 3: 1.9 mm posterior to bregma, 3.0 mm lateral to bregma and 2.0 mm below the skull surface) using a cannula (28-gauge, inner diameter 0.18 mm, outer diameter 0.36 mm) at a rate of 0.2 μl/min. All injection points were to the right hemisphere i.e., the ipsilateral hemisphere. After completion of injection at each point, the cannula was held in place for 10 min before withdrawal.

For *in vitro* studies, the neurons were transduced with the lentivirus at a multiplicity of infection (MOI) of 10 at six days according to the manufacturers’ instruction manual. The oxygen-glucose deprivation (OGD) model and GAL treatment were carried out 3 d after transduction.

### Primary Cortical Neuron Culture and in vitro 1 h OGD/24 h Reoxygenation model

The primary cultured cortical neurons were obtained from postnatal 24 h WT or cPKCγ knockout C57BL/6J mice. Cortical neurons were dissociated with 0.25% trypsin and seeded onto plates at a density of 5×10^5^ cells/cm^2^. Cortical neurons were cultured in Neurobasal Medium with 2% B-27 supplement and 0.25% Glutamax (Gibco Inc, Carlsbad, CA 92008, USA). Half the medium was changed every 72 h.

In the 1 h OGD/24 h reoxygenation model that simulated ischemia-like condition *in vitro*, the culture medium was changed to glucose-free Dulbecco’s Modified Eagle Medium (DMEM). The culture plates were placed in a 37°C anaerobic chamber (Thermo Electron LED GmbH, Langenselbold, Germany) under hypoxic conditions (1% O_2_/5% CO_2_/94% N_2_) for 1 h, and then the primary cultured cortical neurons were maintained in growth culture medium under normoxic condition (21% O_2_/5% CO_2_/74% N_2_) for 24 hr reoxygenation. Treatment with either GAL or AR-M1896 was conducted after the medium was changed for 15 min under normoxic conditions before transferring the plates to the hypoxic chamber.

### Cell Viability Test

To evaluate the effect of GAL on the survival rate of primary cultured cortical neurons undergoing OGD, we planted the neurons at a density of 2 × 10^4^ cells/well in a 96-well plate after dissociation and cell counting. The overall culture method and OGD model were the same as other *in vitro* experiments. On the 7^th^ day, the neurons were exposed to a series of different concentrations of GAL (H-7450, Bachem, Bubendorf 4416, Switzerland) and AR-M1896 (#2699, Tocris Cookson Ltd., Bristol BS110QL, UK) diluted in glucose-free DMEM (10^-13^ to 10^-5^M) and incubated under normoxic conditions for 15 min before being transferred to the hypoxic chamber. After 1 h OGD and 24 h reoxygenation, the Cell Titer 96 Aqueous One Solution Cell Proliferation Assay (Cat. # G3580, Promega, Wisconsin 53711, USA) was used according to the manufacturer’s instructions to determine the overall survival rate of the neurons.

In addition to this form of testing, the CytoTox 96 Non-Radioactive Cytotoxicity Assay (Promega, Wisconsin 53711, USA) was also used to measure lactate dehydrogenase (LDH) release within the culture medium for quantifying cell death. After OGD and reoxygenation, a portion of the neurobasal medium for treated neurons were collected to determine the Experimental LDH Release, and then the lysis buffer that was contained inside the kit was used to induce Maximum LDH release. After incubating the assay plates for 30 min with the assay’s substrate mix, a stop solution was added and absorbance was recorded with an iMark™ Microplate Absorbance Reader (Bio-Rad, Hercules, CA 94547, USA) at 490 nm. Cytotoxicity was calculated using the formula provided in the user’s manual, Experimental LDH Release (OD_490_)/Maximum LDH Release (OD_490_) × 100%. All data were normalized by a control group and presented as cell death rates.

### Caspase Activity Assays

For detection of Caspase activity, we used a series of assay kits from Promega (Madison, WI 53711, USA) on the primary cultured cortical neurons. All of the assays mentioned in this section were performed using the iMark™ Microplate Absorbance Reader (Bio-Rad Laboratories, Hercules, CA 94547, USA).

For detection of Caspase-3 activity, neurons were planted on a 10-cm plate at a density of 5 × 10^5^/cm^2^. After 1 h OGD and 24 h reoxygenation, cell samples were extracted using the lysis buffer included in the CaspACE™ colorimetric assay system (Cat. # G7220, Promega, Madison, WI 53711, USA). The assay was performed in a clear 96-well plate according to the manufacturer’s instructions, with the results presented as a fold ratio of the control.

For detection of Caspase-9 activity, neurons were plated on a white flat-bottom polystyrene 96-well plate. The Caspase-Glo 9 assay system (Cat. # G7211, Promega, Madison, WI 53711, USA) was used according to the manufacturer’s instructions, and the resultant luminescence signal was detected with SpectraMax i3 (Molecular Devices, Sunnyvale, CA 94089, USA).

### Quantitative and Statistical Analysis

Quantitative analysis of immunoblotting results was done through the Quantity-One software (Gel Doc 2000 imaging system, Bio-Rad, Hercules, CA 94547, USA). For protein levels, the ratio of GAL (band density of target protein/band density of β-actin) in the control or sham group was expressed as 1, and the other groups were expressed as fold changes to the normalized control or sham groups. The proteolytic levels of Caspases and LC3 were first calculated into cleavage rates: cleaved Caspase/total Caspase or LC3-II/(LC3-II + LC3-I), and then normalized by their respective control or sham groups. All values are represented as mean ± SEM. Statistical analysis was performed using one-way analysis of variance (ANOVA) followed by the Bonferroni multiple comparison test with the software GraphPad Prism version 6.00 for Mac (GraphPad Software, La Jolla, CA 92037, USA). A p value of less than 0.05 was regarded as significant.

**Figure 1.**
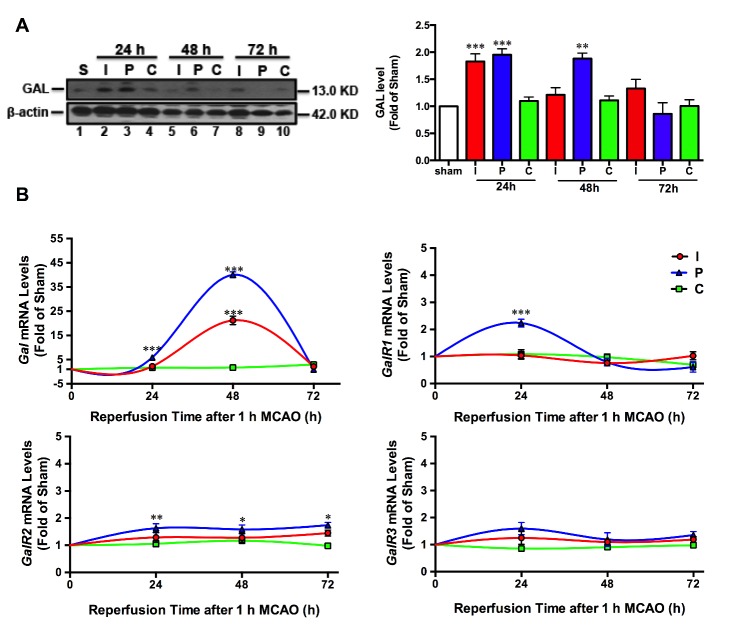
**Dynamic changes of GAL protein, *GAL* mRNA and its receptors *GalR1-3* mRNA expression levels after ischemic stroke. A)** Representative Western blot and quantitative analysis showing GAL protein expression levels in the infarct core (I), peri-infarct region (P) and contralateral cortex (C) of mice after 1 h MCAO/24-72 h reperfusion-induced ischemic stroke (n=6 per group). **B)** Relative gene expressions of *GAL* mRNA and its receptors *GalR1-3* mRNAs in the I, P and C regions of mice after 1 hr MCAO/6-72 h reperfusion (n=6 per group). Data are presented as mean ± SEM; *p<0.05, **p<0.01, ***p<0.001 *vs.* sham.

## RESULTS

### Changes of GAL peptide and GAL/GalRs mRNA expressions after ischemic stroke

After 1 hr MCAO-induced ischemia, mice were euthanized after 24, 48 and 72 hr of reperfusion. The typical Western blot and quantitative analysis results (Fig. 1A) showed that GAL expressions were significantly increased after 24 (*p*=0.0003) and 48 hr (*p*<0.001) of reperfusion in the peri-infarct region (P); there was also a significant increase of GAL protein expression in the infarct core (I) after 24 hr of reperfusion (n=6 per group). Unlike the trend in the GAL peptide, the *GAL* mRNA expression in both the infarct and peri-infarct regions peaked at 48 hr after reperfusion (*p*<0.001). As for GAL receptors, both *GalR1* and *GalR2* (but not *GalR3*) mRNA expressions were significantly increased (*p*<0.001 for *GalR1*, *p*=0.0087 for *GalR2*) in the peri-infarct region after 24 hr of reperfusion. Further, *GalR2* mRNA expression was slightly increased in the peri-infarct region after 48 hr (*p*=0.0189) and 72 hr (*p*=0.0292) of reperfusion (n=6 per group; Fig. 1B).

**Figure 2.**
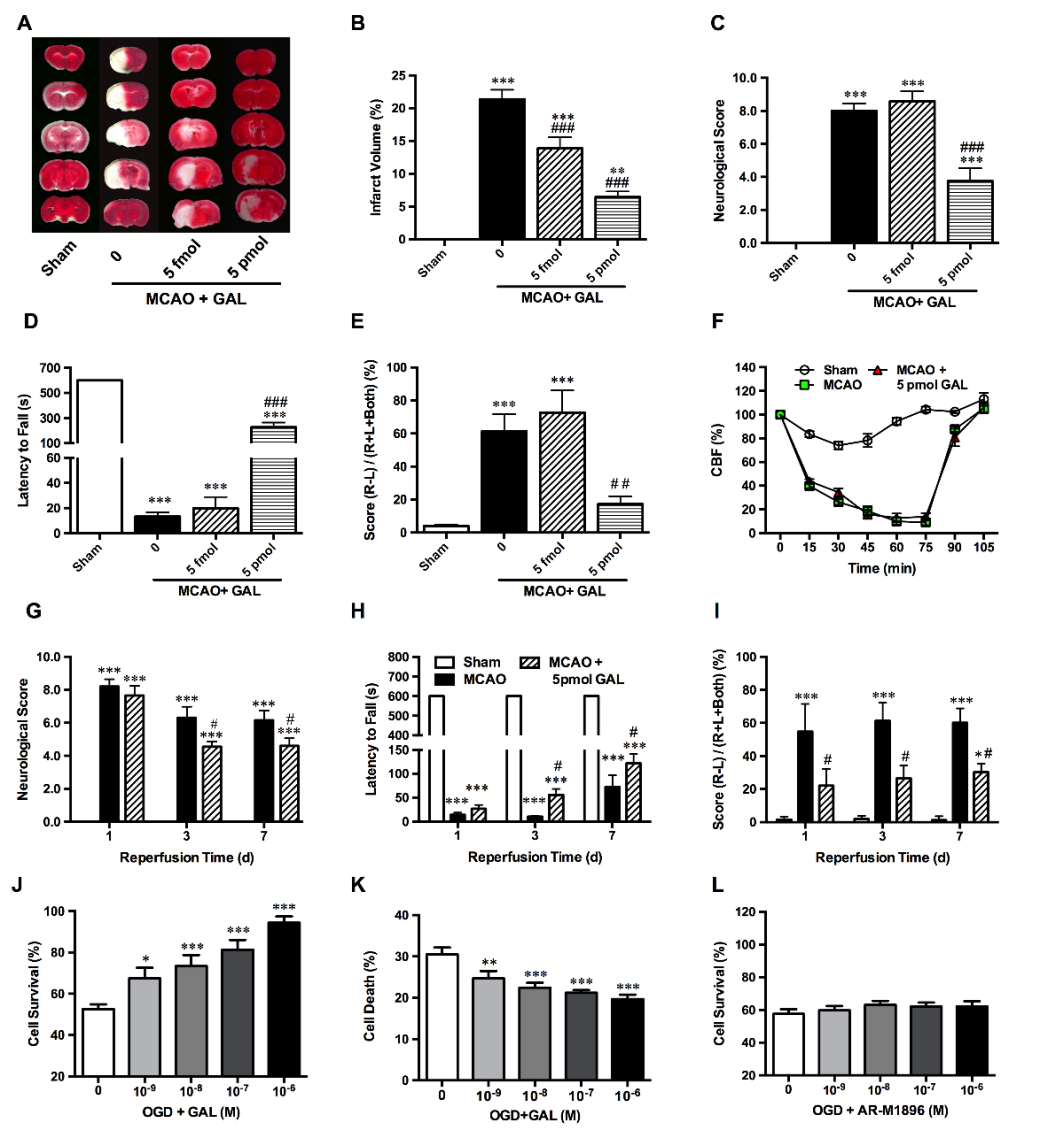
**Galanin treatment protects neurons against MCAO and OGD-induced ischemic injuries. (A and B)** Representative TTC staining and graphs, showing significant decrease of infarct volume of ischemic stroke mice after 5 fmol and 5 pmol GAL pre-treatment (n=6 per group); **(C)** Neurological scores of mice after 1 h MCAO and 24 h reperfusion showed a significant attenuation of deficits at 5 pmol (but not 5 fmol) GAL pre-treatment (n=8 per group); **(D)** Evaluation of muscle strength using the wire hanging test. All sham group mice could stay on the wire mesh for the maximum observation period of 600s, but MCAO mice showed a significant decrease of latency time. 5 pmol GAL pre-treatment also showed a significant improvement, but still significantly lower than the sham group (n=8 per group); **(E)** Evaluation of limb coordination via the cylinder test shows a marked increase of contralateral limb usage in mice receiving 5 pmol GAL pre-treatment (n=8 per group); **(F)** Cerebral blood flow (CBF) of the middle cerebral artery detected via Laser Doppler flowmetry, which shows a significant decrease of CBF after insertion of the intraluminal monofilament, but no changes were observed after the i.c.v injection of GAL (n=5 per group); **(G)** Neurological scores of mice after 1 hr MCAO/3-7 d reperfusion showed a significant attenuation of deficits after 5 pmol GAL post-treatment (n=8 per group); **(H)** Evaluation of muscle strength via the wire hanging test. MCAO mice showed a significant decrease of latency time. Conversely, the 5 pmol GAL post-treatment group showed a significant improvement (n=8 per group); **(I)** Evaluation of limb coordination the cylinder test, showing a marked increase of contralateral limb usage of ischemic stroke mice receiving 5 pmol GAL after reperfusion (n=8 per group); **(J)** The MTS assay results show that pre-incubation with GAL 15 min prior to OGD could increase neuronal survival rates in a dosage dependent manner in primary cultured cortical neurons after 1 h OGD/24 hr reoxygenation (n=6 per group); **(K)** The cytotoxicity assay results indicate a significant decrease of cell death rates in GAL-treated group when compared with OGD group (n=6 per group); **(L)** MTS assay demonstrate that pre-incubation with the GalR2/3 agonist AR-M1896 at various concentrations 15 min prior to OGD did not affect neuronal survival rates of primary cultured cortical neurons after 1 h OGD/24 h reoxygenation (n=6 per group). Data are presented as mean ± SEM; *p<0.05, **p<0.01, ***p<0.001 *vs.* sham/0 M GAL; #*p*<0.05, ##p<0.01, ###*p*<0.001 *vs.* MCAO group.

**Figure 3.**
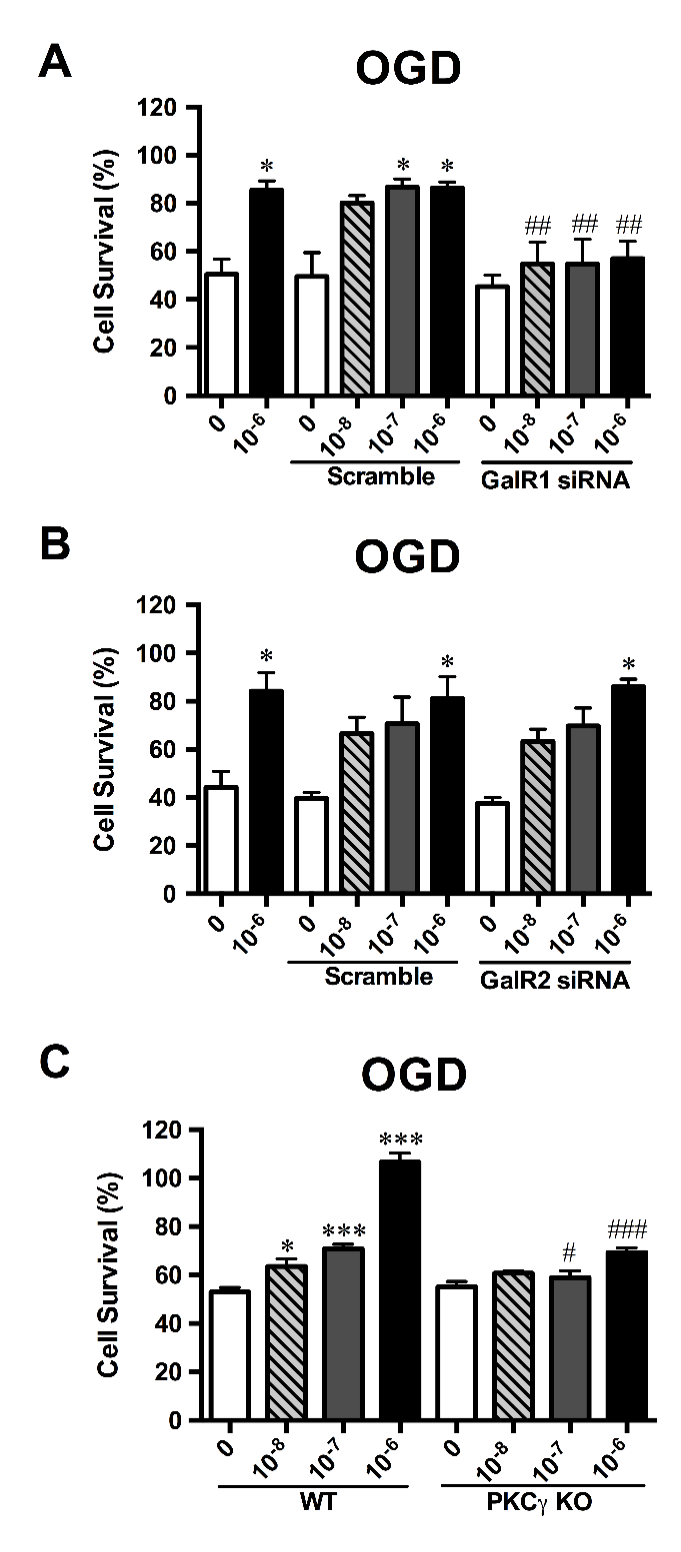
**Galanin’s neuroprotective properties are dependent on GalR1 and cPKCγ regulation in primary cultured cortical neurons.** Utilizing siRNA knockdown in primary cultured cortical neurons, GAL’s protective effects were negated with GalR1 knockdown **(A)** while GAL’s effects remained unchanged in GalR2 knockdown neurons **(B)**, n=18 per group; Using primary cultured cortical neurons derived from neonatal cPKCγ KO mice, we found that GAL was unable to extend its neuroprotection to OGD-treated neurons without the downstream signaling of cPKCγ **(C)**, n=18 per group. Data are presented as mean ± SEM; *p<0.05 *vs.* 0 M GAL; ^#^*p*<0.05, ^##^*p*<0.01, ^###^*p*<0.001 *vs.* corresponding GalR Scramble/cPKCγ WT.

### Effects of GAL treatment on functional outcomes after ischemic stroke and survival rate of primary cultured cortical neurons after 1 h OGD/24 hr reoxygenation

To determine the effects of GAL, GAL was administered 1 h before MCAO surgery via i.c.v injection. After 24 hr reperfusion, behavioral tests were carried out before assessment of infarct volume with TTC staining. We found that GAL at concentrations of both 5 fmol and 5 pmol significantly decreased infarct sizes (*p*=0.0146 for 5 fmol and *p*<0.001 for 5 pmol; n=6 per group; Figs. 2A and B). Yet, only the higher concentration (5 pmol) had an effect on the outcome of behavioral tests, showing significantly decreased neurological deficits (*p*=0.8518 for 5 fmol and *p*<0.001 for 5 pmol; n=8 per group; Fig. 2C), improved motor coordination in cylinder tests (*p*=0.7721 for 5 fmol and *p*=0.0033 for 5 pmol; n=8 per group; Fig. 2D) and increased muscle strength (*p*=0.9903 for 5 fmol and *p*<0.001 for 5 pmol; n=8 per group; Fig. 2E). In order to eliminate GAL’s effects on metabolism and blood flow, mice body weights were monitored daily (data not shown) while Laser Doppler flowmetry was used to monitor regional cerebral blood flow. Both tests showed negative results when comparing the GAL-treated to MCAO groups, suggesting that GAL’s protective effects in acute cerebral ischemic injury was not the result of changes in body weight or intensified blood flow in the infarct regions (n=5 per group; Fig. 2F), but could be attributed to another underlying mechanism.

In addition, for a more clinically relevant comparison that has the potential for future translational research, we tested the effects of GAL post-treatment after 6 h reperfusion. Behavioral studies on mice injected with 5 pmol GAL show similar effects as that of mice in pre-treatment groups that showed decrease in neurological deficit scoring at 3 (*p*=0.0141) and 7 (*p*=0.0337) days after reperfusion (n=8 per group; Fig. 2G), increased muscle strength on wire hanging tests at 3 d (*p*=0.0198) and 7 d (*p*=0.01) after reperfusion (n=8 per group; Fig. 2H), and improved forelimb coordination at 1 d (*p*=0.0254), 3 d (*p*=0.0158) and 7 d (*p*=0.0445) after reperfusion in cylinder tests (Fig. 2I)

Furthermore, a single dose of GAL was given to 1 h OGD/24 h reoxygenated-treated primary cultured cortical neurons at concentrations ranging from 10^-9^ to 10^-5^ M for 15 min prior to subjecting them hypoxia. The MTS assay results showed that GAL could increase the survival rate of OGD-treated neurons in a dosage-dependent manner (n=6 per group; Fig.2J), its effects starting to take place at 10^-9^ M (*p*=0.0385), with a stark contrast at 10^-8^ M (*p*=0.0007), 10^-7^ M (*p*<0.001), 10^-6^ M (*p*<0.001), and 10^-5^M (*p*<0.001). When fitted into a dose-response curve, the log_EC50_ was calculated as -7.382. To corroborate this finding, we saw a significant decrease in Lactate Dehydrogenase (LDH) release rates, e.g. cell death, in neurons treated with GAL. Similar to the MTS assay results, this effect was also in a dosage-dependent manner, during which the peptide started to take effect at 10^-9^ M (*p*=0.0216), with stronger effects at higher levels of 10^-8^ M (*p*<0.001), 10^-7^ M (*p*<0.001) and 10^-6^ M (*p*<0.001) (n=6 per group; Fig. 2G). However, AR-M1896, a GalR2/3 agonist did not have the same protective effect (n=6 per group; Fig. 2H).

**Figure 4.**
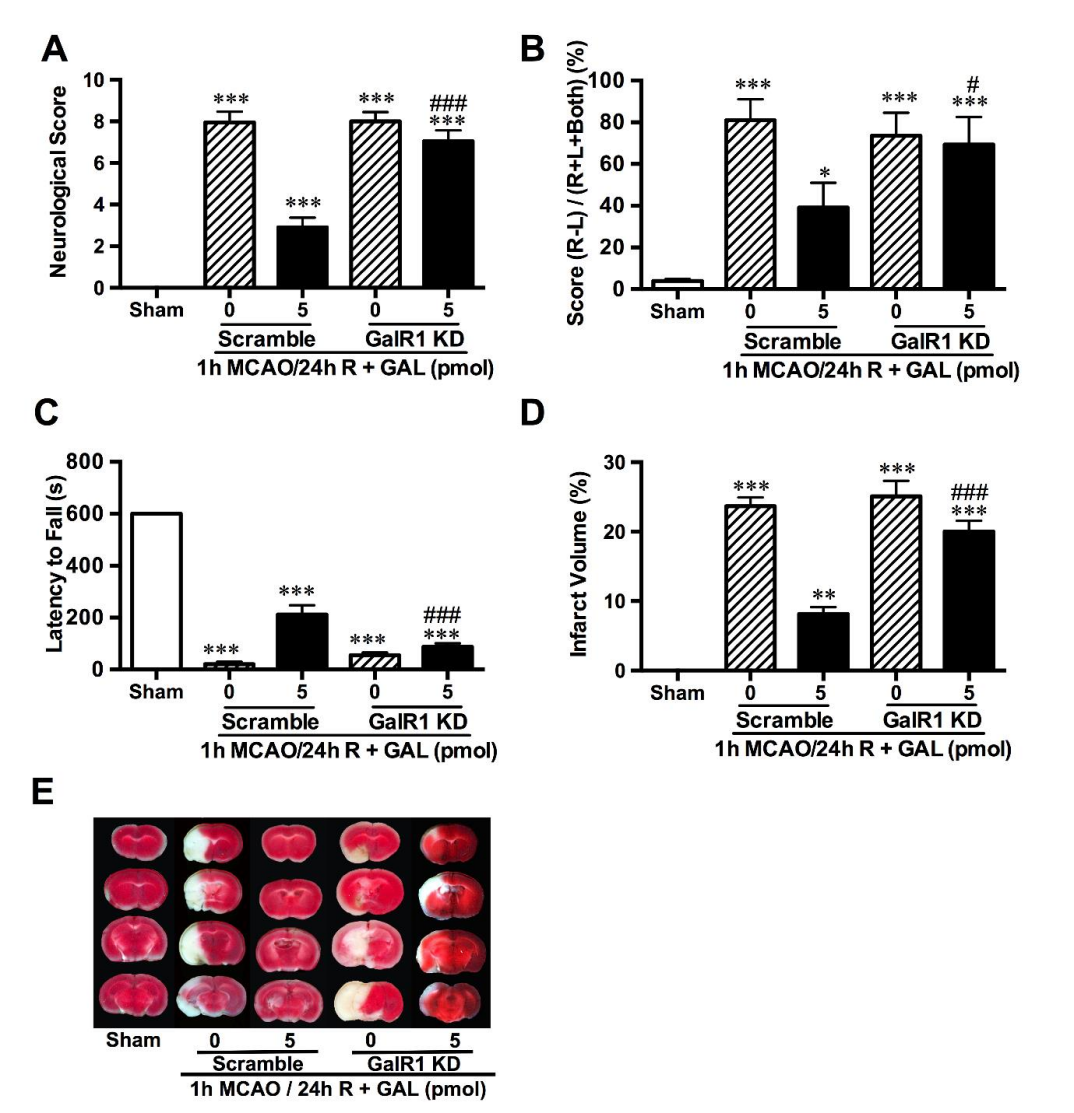
**Galanin-mediated neuroprotection against ischemic injury is dependent on GalR1.** In animal models, GalR1 knockdown (KD) prior to GAL injection and MCAO was able to negate the attenuation of neurological deficits caused by GAL. The overall neurological deficit score **(A)** of GalR1 KD remained on par with that of the MCAO group, while limb coordination **(B)** and muscle strength **(C)** failed to show marked recovery when compared to the scramble + GAL-treated group. **E and D)** Representative photos and statistical analysis of TTC staining, showing negation of GAL’s protection after GalR1 knockdown. Data are presented as mean ± SEM; **p<0.01, ***p<0.001 *vs.* sham; ^#^*p*<0.05, ^###^*p*<0.001 *vs.* corresponding GalR1 Scramble+5 pmol treated MCAO group; n=8 per group.

**Figure 5.**
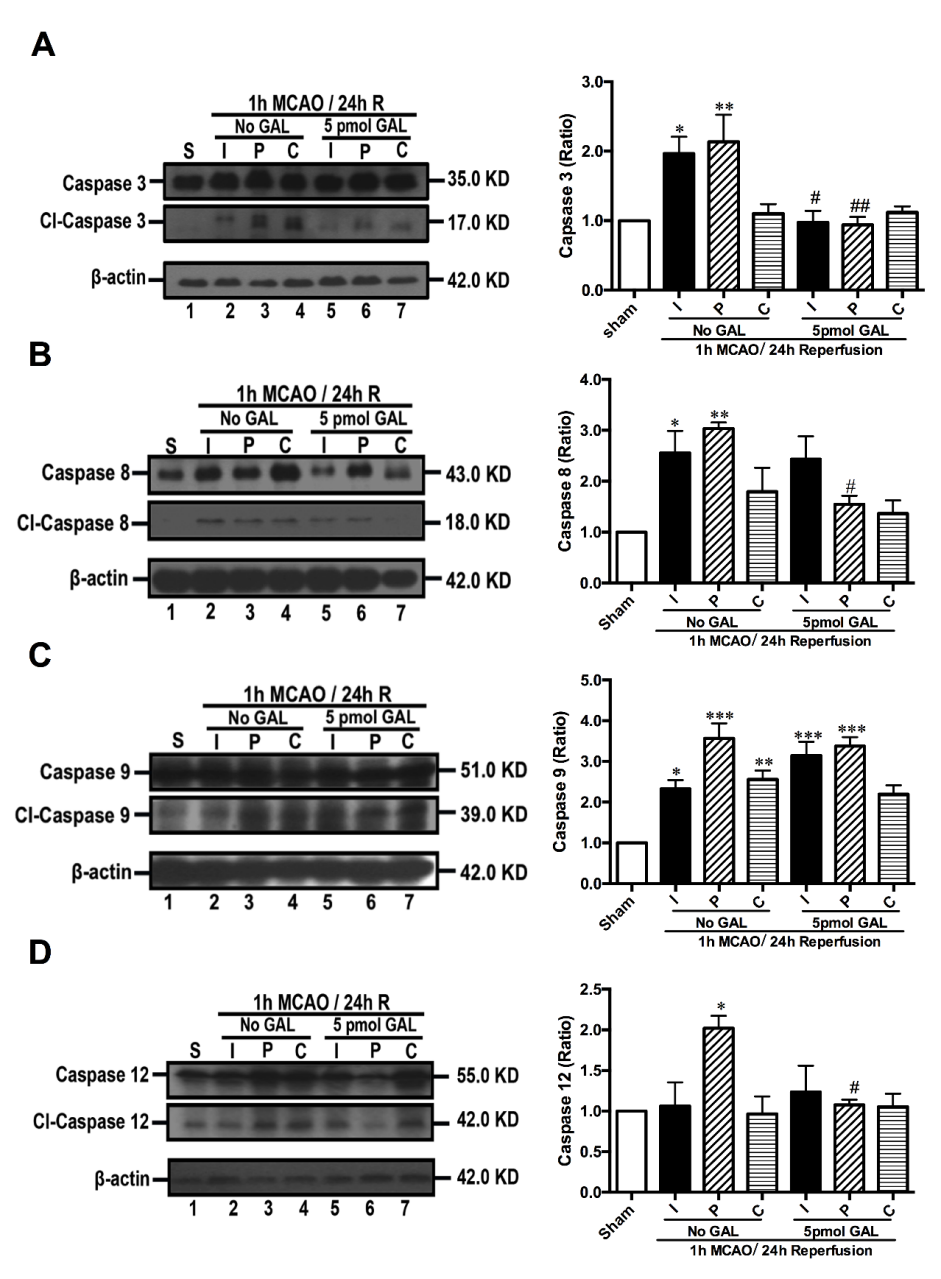
**Galanin inhibits Caspase activity in the peri-infarct region of mice with ischemic stroke.** After isolating the infarct core (I), peri-infarct region (P) and contralateral cortex (C) of mice following 1 h MCAO/24 h reperfusion and GAL treatment, Western blotting results demonstrate that the cleaved-Caspase/Caspase ratios of Caspase-3 **(A)**, Caspase-8 **(B)**, and Caspase-12 **(D)** decreased significantly, but Caspase-9 **(C)** did not show significant changes in the 5 pmol GAL treatment group when compared with the MCAO group (n=6 per group). Data are presented as mean ± SEM; *p<0.05, ** *p*<0.01, *** *p*<0.001 *vs.* sham; ^#^*p*<0.05, ^##^*p*<0.01 *vs.* MCAO group.

### GAL’s protective effects were abolished by GalR1 knockdown and cPKCγ knockout

In order to find a potential signaling pathway that GAL might work through to exert its protective effects, the effects of GAL was accomplished with knockdown of specific receptor subtypes. Using the MTS assay to assess cell survival rate, neither GalR1 siRNA nor its scramble affected the cell viability of OGD-treated neurons under normoxic conditions. However, GAL’s protective effects were abolished when GalR1 was knocked down in OGD-treated neurons (n=18 per group; Fig. 3A), suggesting that GalR1 is involved in mediating GAL’s protective effects. In order to determine whether manipulating GalR2 expression levels in primary cultured neurons would affect GAL’s role in ischemic stroke, lentiviral knockdown of GalR2 in primary cultured neurons was utilized. Upon knockdown of GalR2, neurons did not show any change in survival rate under normoxic conditions nor were GAL’s neuroprotective abilities on OGD-treated neurons impaired (n=18 per group; Fig. 3B). Taken together, it is very possible that GAL exerts neuroprotection through GalR1 and not GalR2.

Moreover, behavioral studies and TTC staining showed that after GalR1 knockdown, GAL did not have significant effects on the level of neurological deficits in the MCAO group (*p*<0.001 when compared with sham; *p*=0.5384 when GalR1 KD-0 *vs.* GalR1 KD-5 pmol; n=8 per group; Fig. 4A). However, when the mice received a scrambled version of the siRNA i.e., scramble (sc), GAL still had a neuroprotective effect, which effectively lowered the deficits (sc-0 *vs.* sc-5 pmol, *p*<0.001; Fig. 4A). This phenomenon was also observed in cylinder tests, in which the forelimb coordination of mice was severely encumbered after MCAO (for GalR1 KD-0, GalR1 KD-5 and sc-0, *p*<0.001 when compared to sham, for sc-5 pmol *vs.* sham *p*=0.0475; n=8 per group; Fig. 4B). GalR1 KD mice showed no signs of attenuation even when treated with GAL (GalR1 KD-0 *vs.* GalR1 KD-5 pmol, *p*=0.9991; Fig. 4B), yet the GAL-treated mice that received the scramble had significantly milder forelimb deficits than the untreated mice (sc-0 *vs.* sc-5 pmol, *p*=0.0272, Fig. 4B). In the wire hanging test, all MCAO groups had reduced muscle strength when compared to sham and GAL treatment failed to prolong the latency to fall time (when compared to sham *p*<0.001; for GalR1 KD-0 *vs.* GalR1 KD-5 pmol, *p*=0.7179; n=8 per group; Fig. 4C), but in the scrambled sequence groups, GAL treatment showed a significantly longer time of holding to the wire grid (sc-0 *vs.* sc-5 pmol, *p*<0.001; Fig. 4C). TTC staining also showed that GalR1 KD abolished the protective effects of GAL (GalR1 KD-0 *vs.* GalR1 KD-5 pmol, *p*=0.1108; n=8 per group; Figs. 4D and E).

Since conventional protein kinase C (cPKC)γ has been reported to play a key role in endogenous protective mechanisms [20, 21], and association between GalR1 and protein kinase C (PKC) has also been found in the cardiovascular system [30], we hypothesized that cPKCγ could be involved in mediating the GAL-induced neuroprotection that was observed. By using primary neurons extracted from cPKCγ KO mice, we found that GAL’s protection during 1 h OGD/24 h reoxygenation is no longer effective without the involvement of cPKCγ (PKCγ KO 0 *vs.* PKCγ KO 10^-6^ M GAL, *p*=0.1094; n=18 per group; Fig. 3C); there was a significant decrease in cell survival rates between wildtype (WT) and cPKCγ KO neurons after GAL treatment (WT+10^-6^ M GAL *vs.* PKCγ KO+10^-6^ M GAL; *p*<0.001). However, cPKCγ KO does not seem to affect neuronal viability under normoxic conditions.

### GAL treatment decreased neuronal apoptosis after ischemic injury

**Figure 6.**
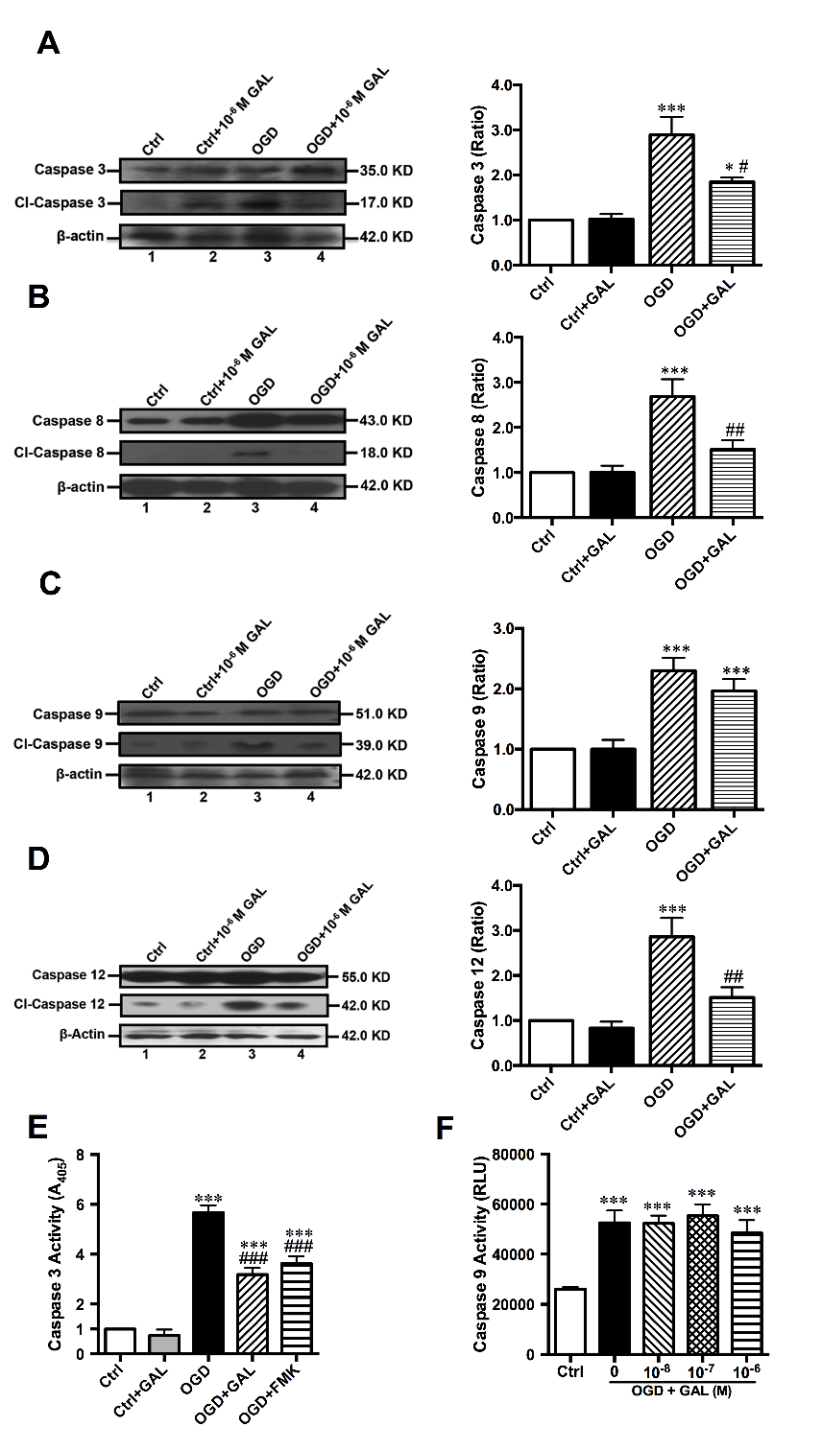
**Galanin inhibits Caspase activity in neurons after 1 h OGD/24 h reoxygenation. A)** Representative and statistical analysis of Western blot results on Caspase-3 in primary cultured cortical neurons after 1 h OGD/24 h reoxygenation, showing a significant decrease of cleaved Caspase-3 in the GAL treatment group (n=6 per group). **B)** Western blot results for Caspase-8 showed a significant decrease of cl-Caspase-8/Caspase-8 ratio in GAL-treated neurons (n=6 per group); **C)** Western blot results for Caspase-9 showed a marked increase of cl-Caspase-9/Caspase-9 ratio in OGD-treated cortical neurons, but no difference was observed between OGD and OGD+GAL treatment groups (n=6 per group). **(D)** Western blot results for Caspase-12 showed a significant decrease of cl-Caspase-12/Caspase-12 ratio in OGD+GAL treated neurons (n=6 per group). **E)** Assessment of Caspase-3 activity in neurons using the CaspACE Assay System, showing a significant decrease in the GAL treatment group (n=6 per group). **F)** Assessment of Caspase-9 activity via the Caspase 9-Glo Assay kit. A significant increase in Caspase activity was observed in OGD groups when compared with control, but no significant difference was seen between the OGD+GAL and OGD-treated groups (n=12 per group). Data are presented as mean ± SEM; *p<0.05, *** *p*<0.001 *vs.* control; ^#^*p*<0.05, ^##^*p*<0.01, ^###^*p*<0.001 *vs.* OGD group.

In order to explore the mechanisms behind this protective effect of GAL, we used Western blotting to assess the expression levels of Caspase-3 in tissue samples extracted from the brains of MCAO mice and those treated by GAL. We found a significant decrease of Caspase-3 cleavage in both the infarct core (*p*=0.0151) and the peri-infarct area (*p*=0.0017; n=6 per group; Fig. 5A). This decrease of Caspase-3 cleavage was also observed in Western blot results of OGD-treated primary cultured cortical neurons (*p*=0.0071; n=6 per group; Fig. 6A). Using the CaspACE™ Colorimetric Assay, we confirmed a marked reduction of Caspase-3 activity in neurons treated with 10^-6^ M GAL (*p*<0.0001; n=6 per group; Fig. 6E).

To further examine GAL’s effects on initiator Caspases, we used immunoblotting on animal tissue to determine whether GAL could change the cleavage levels of Caspases-8, -9 and -12. We found that the levels of cleaved Caspase-9 (*p*=0.9993; n=6 per group; Fig. 5C) remained unchanged while both Caspase-8 and Caspase-12 saw significant decreases in their cleavage (*p*=0.0456 for Caspase-8 and *p*=0.0336 for Caspase-12; n=6 per group; Figs. 5B and D) in the peri-infarct region of MCAO mice after GAL treatment. The *in vitro* OGD models also demonstrated a significant decrease of cleaved Caspase-8 and -12 levels in the GAL treatment group (*p*=0.0037 and *p*<0.01, respectively; n=6 per group; Figs. 6B and D) in primary cultured cortical neurons. The insignificant expression level of Caspase-9 was also corroborated by *in vitro* experiments (*p*=0.4797; n=6 per group; Fig. 6C) as well as the Caspase-Glo 9 Assay (*p*>0.9999; n=12 per group; Fig. 6F). These results suggest that a possible mechanism of GAL’s neuroprotective effects is the inhibition of Caspase-8 and -12-initiated apoptosis.

## DISCUSSION

Using an intraluminal MCAO model in the mouse, we demonstrate for the first time that galanin is neuroprotective in ischemic stroke. Moreover, our data indicate that GalR1 is the receptor subtype responsible for GAL’s protective effects and a possible mechanism is the inhibition of Caspase-8 and Caspase-12-initiated apoptosis via the cPKCγ pathway.

As a neuropeptide, GAL has been known to be involved in several prominent processes of the nervous system, such as memory function, regulation of hypothalamopituitary hormones, seizure activity, neuropathic pain, inflammation and neuroprotection [31]. GAL has been reported to have neuroprotective roles in several cases, such as traumatic brain injury [32], beta-amyloid poisoning [11-13], glutamate induced excitotoxicity [14], high-glucose induced apoptosis and shear stress induced injury [15, 16]. However, its role in ischemic stroke remains unclear. Here, we provided evidence for the first time that GAL can protect the mouse brain from ischemic stroke injury resulting not only in a reduced infarct size, but also alleviating neurological deficits. However, an earlier study by Holm et al looked at the effects of GAL via continuous intracerebroventricular infusion and did not find any changes in the infarct area between GAL-treated and MCAO only groups [33]. The reasons for the inconsistency could be: 1) different experimental stroke models used, an intraluminal MCAO model was used in the present study while a craniotomy MCAO model was performed in the earlier study; 2) different neuropeptide delivery methods, a single injection of freshly prepared GAL was applied in the present study, but a continuously infused i.c.v was applied in the earlier study; and 3) timing of TTC staining, due to the inert limitations of TTC staining, this method cannot provide a reliable delineation of the infarct core beyond 24 h [34]. Moreover, our *in vitro* experiments also showed that GAL protected neurons in a dose dependent manner with an IC_50_ of 10^-7^ M in the *in vitro* OGD model. GAL is a multi-talented neuropeptide that not only plays a neuroprotective role during CNS injury, but is also involved in other pathophysiological processes, suggesting that GAL could target multiple pathways in the progressive cell death following ischemic injury. In addition, GAL is an endogenously expressed neuropeptide, so it would have a better chance to act on patients without the common side effects of pharmaceutical drugs.

Although i.c.v injection before MCAO is not the most clinically relevant method of drug administration, the limitations of post-surgery care and high lethality of ischemic stroke makes it a more plausible route during initial studies of basic research. In fact, we also observed a significant improvement in neurological outcome after GAL post-treatment in ischemic stroke mice at 1-7 d. In addition, several studies point towards ischemic/hypoxic preconditioning as a method for activating the body’s endogenous protective mechanisms, such as attenuation of infarct and blood-brain barrier (BBB) disruption via remote ischemic preconditioning [35]. Recently, other discoveries have also seen the reduction of ischemic injury by pretreating with a Cannabinoid Receptor 2 agonist or Warfarin [36, 37]. Consequently, in our future studies, we will look for a safer administration route that is equally effective, such as intranasal injection, which has been shown to have positive effects when treating stroke patients with insulin and insulin growth factor 1 [38]. In addition, the intranasal administration method has also been shown to be effective when treating obese mice with GAL-like peptides [39]. Meanwhile, after 24 h of reperfusion, both GAL and GalR1 showed a significant increase in the ipsilateral versus contralateral cortex, suggesting GAL’s involvement in ischemic stroke. In fact, GAL mRNA expression rose abruptly after 24 h of reperfusion and reached a peak of 20-fold its original after 48 h reperfusion. All these increases might play a role of endogenous protection in the early stages of ischemic stroke.

GAL acts upon its three-receptor subtypes termed as Gal-R1, -R2 and -R3 [10]. It has been reported that the GalR2 signaling pathway mainly mediates the protective/trophic effect of GAL [14, 40]. However, in the present study, this protection effect was blocked by RNAi knockdown of GalR1, but not GalR2. Meanwhile, the agonist of GalR2 and GalR3, AR-M1896 [41], has no effect on the GAL’s protective effects, suggesting that GalR1 might be the main receptor subtype mediating the effect of exogenously administered GAL. Moreover, RT-qPCR results showed that GalR1 mRNA expressions was increased in the peri-infarct region, which is consistent with an earlier study that demonstrated a significant increase of GalR1 in the peri-infarct region 24 h after transient focal cerebral ischemia [17]. Due to the complexities of the GalR structure and lack of receptor subtype-specific agonists and antagonists, AR-M1986 is probably the most specific agonist that Galanin receptors currently, and even this ligand has been found to activate both GalR2 and GalR3 [10] thus, the only specific alternate approach that was available is knockdown via siRNA. Perhaps with further developments in the area of receptor subtype-specific agonist or antagonist for GalR1, we will finally be able to confirm our findings with a GalR1-specific agonist or antagonist. In addition, we plan to utilize GalR1 knockout and mice that overexpress GalR1 to further determine GAL’s molecular mechanism in future studies, which is also the reason why mice were used as test subjects in the present study.

Being a ubiquitously expressed protein, the PKC family of serine/threonine kinases has been found to be present in several body systems and its activity seen in multiple tissues during ischemic injury [42-45]. It has been reported that cPKCγ saw a significant increase as high as 24 fold in tissue samples obtained from the penumbra of ischemic stroke patients [46]. Moreover, we have also shown that cPKCγ is a key factor in ischemic stroke injury and plays an important role in the brain’s endogenous protective pathways during preconditioning [20]. In the present study, *in vitro* experiments showed that cPKCγ KO compromised GAL’s ability to increase the survival rate of neurons during OGD, indicating that cPKCγ is involved in GAL protective mechanisms. However, the link between GalR1, cPKCγ, and the downstream mechanisms, which regulate this neuroprotection in ischemic stroke injury, remains to be explored. In addition, PKC is a family of serine/threonine kinase comprised of 10 isoforms, with differences in requirement of Ca^2+^ and phospholipids for activation [47]. Although this study shows that cPKCγ plays a key role in the downstream signaling of GAL’s protective effects, further studies are needed to determine the involvement of other isoforms.

Studies on the pathogenesis of ischemic stroke show apoptosis as a key player in the ischemic core and penumbra [48]. Here we found that one of the mechanisms utilized by GAL to extend its protective effects is the inhibition of Caspase-8/-12-initiated apoptosis. This is consistent with earlier studies that silencing GalR1 could induce apoptosis in cancer cells [49]. In order to determine the specific pathway that GAL activates, we also investigated the cleavage of Caspase-8, -9 and -12, which represent the main initiators of the death receptor pathway, the mitochondria pathway and endothelium reticulum (ER) stress, respectively. All of which have been reported to be involved with ischemic injury. Findings from Western blot analysis showed that GAL could decrease the cleavage rate of both Caspase-8 and -12, but does not affect the expression levels of cleaved Caspase-9.

Besides apoptosis, ischemic stroke can also induce other types of cell death, such as autophagy and cytotoxicity caused by excess levels of reactive oxygen species (ROS) and oxidative stress [50]. Upon detection of autophagy-related proteins via immunoblotting, we found that GAL treatment enhanced LC3 (microtubule-associated protein 1 light chain 3) proteolysis and Beclin 1 expression levels (data not shown), suggesting that in addition to inhibiting apoptosis, GAL could also increase autophagy in the peri-infarct region of mice that underwent 1 h MCAO/24 h reperfusion. However, recent studies on autophagy have emphasized the importance of the autophagy flux, which is the process of cargo sequestration in autophagosomes to their delivery and degradation in lysosomes, and the serious consequences that disrupted autophagy flux could have on neurons, especially during CNS injury and ER stress [51, 52]. In addition, increase of LC3-II accumulation could be due to either an increase of autophagosome formation or disruption in its degradation [53]. Although increases of Beclin 1, which is associated with autophagy initiation, seems to indicate increase of autophagosome formation. Further studies are needed to determine the progress of the autophagy flux and whether it is a sign of neuroprotection or a response to ischemic injury. Furthermore, *in vitro* findings with the ROS-Glo H_2_O_2_ assay kit (Promega) showed that GAL treatment did not affect H_2_O_2_ release rates in primary cultured cortical neurons after 1 h OGD/24 h reoxygenation (data not shown).

In conclusion, we first provide evidence that GAL protein and mRNA show significant increases within 48 h of reperfusion after 1 h MCAO, establishing a possible connection between GAL and the mouse brain under ischemic stroke. We then provided evidence that GAL can reduce neuronal damage and improve neurological outcomes after ischemia/reperfusion injury, and one of the potential pathways for GAL to extend its protective effects is through the inhibition of Capsase-8/12-initiated apoptosis. Meanwhile, GalR1 and cPKCγ seemingly play pivotal roles in the downstream signaling of this particular pathway for GAL. Thus, drawing from the evidence presented above, we believe this study could provide us with an exciting new target to study, both in terms of gaining more insight on stroke and in finding a potential drug for treatment.
